# Primary versus postoperative gamma knife radiosurgery for intracranial benign meningiomas: a matched cohort retrospective study

**DOI:** 10.1186/s12885-022-09321-w

**Published:** 2022-02-24

**Authors:** Junyi Fu, Jiamin Zeng, Minyi Huang, Shunyao Liang, Yong He, Longchang Xie, Yinhui Deng, Jinxiu Yu

**Affiliations:** 1 Department of Neurology, The Second Affiliated Hospital of Guangzhou Medical University, Guangzhou, 510260 Guangdong China; 2 Department of Pathology, The Second Affiliated Hospital of Guangzhou Medical University, Guangzhou, 510260 Guangdong China; 3 Department of Radiotherapy, The Second Affiliated Hospital of Guangzhou Medical University, Guangzhou, 510260 Guangdong China

**Keywords:** Gamma knife, Stereotactic radiosurgery, Meningioma, Primary radiosurgery

## Abstract

**Objective:**

The aims of this study were to investigate the long-term outcomes of primary versus postoperative Gamma Knife radiosurgery (GKRS) for benign meningiomas.

**Methods:**

Three hundred and forty meningioma patients underwent GKRS were retrospectively reviewed. Patients in the postoperative GKRS group were matched to those in the primary GKRS group, in a 1:1 ratio.

**Results:**

The study consisted of 122 patients, including primary (*n* = 61) and postoperative (*n* = 61) GKRS group. Thirty-four patients (27.9%) occurred radiological progression after a median follow-up of 72.5 (range, 24.2–254.5) months. The median time to radiological progression was 85.1 (range, 20.7–205.1) months. The radiological progression-free survival (PFS) was 100%, 93%, 87%, and 49%, at 1, 3, 5, and 10 years respectively. Thirty-one patients (25.4%) occurred clinical progression. The clinical PFS was 92%, 89%, 84%, and 60%, at 1, 3, 5, and 10 years. In combined group, only max diameter ≥ 50 mm was associated with radiological (*p* = 0.020) and clinical PFS (hazard ratio [HR] = 2.896, 95% confidence interval [CI] = 1.280–6.553, *p* = 0.011). Twenty-five patients (20.5%) developed GKRS related adverse effects, including radiation-induced edema (*n* = 21). Non-skull base tumors (HR = 3.611, 95% CI = 1.489–8.760, *p* = 0.005) and preexisting peritumoral edema (HR = 3.571, 95% CI = 1.167–10.929, *p* = 0.026) were significantly related to radiation-induced edema in combined group. There was no significant difference in radiological PFS (*p* = 0.403), clinical PFS (*p* = 0.336), and GKRS related adverse effects (*p* = 0.138) between primary and postoperative GKRS groups.

**Conclusions:**

Primary GKRS could provide similar radiological and clinical outcomes, as well as similar complication rate compared with postoperative GKRS. For selective benign meningioma patients (asymptomatic or mildly symptomatic tumors; unfavorable locations for surgical resection; comorbidities or an advanced age), GKRS could be an alternative primary treatment.

## Introduction

Meningiomas are the most common non-malignant intracranial tumors, which account for approximately 37.6% of all intracranial tumors [1]. Of those with the documented WHO grade, 80.5% are grade I [1]. For some benign meningiomas (such as skull base meningiomas), the rate of tumor growth is slow, with the mean tumor volume doubling time of approximately 8 years [2, 3]. However, it is challenging for the treatment of benign meningiomas. If therapy is indicated, surgical resection should be considered the first therapeutic option in meningiomas of all WHO grades [4]. Some patients can be cured by surgical resection alone, especially for benign tumors in favorable locations. For tumors in unfavorable locations (such as skull base tumors and those close to vascular or neural structures), complete surgical resection is complex and may cause serious complications.

Gamma knife radiosurgery (GKRS) is a less invasive treatment with low morbidity, more appealing than surgical resection. In a systematic review, meta-analysis and practice guideline from international stereotactic radiosurgery society [5], stereotactic radiosurgery (SRS) is recommended in the following circumstances: 1) complete resection cannot be achieved or is not amenable; 2) as a primary treatment for asymptomatic or mildly symptomatic meningiomas; 3) for postoperative recurrence or progression tumors. For WHO grade I meningiomas treated with SRS, the progression-free survival (PFS) ranged from 85 to 100% (median, 89%), and from 53 to 100% (median 85%) at 5 and 10 years respectively, with a low rate of toxicity [5]. Several studies reported that prior surgery was related to worse local control [6, 7]. El-Khatib et al. [8] found a better PFS in patients with primary SRS than with adjuvant or salvage SRS. Pollock et al. [9] and Sheehan et al. [10] also found that prior surgery adversely affected tumor control. However, Kim et al. [11] did not find any relationship between prior surgery and tumor control. Up to now, the effectiveness of primary versus postoperative GKRS for benign meningiomas is still controversial. Therefore, in order to compare the outcomes of primary versus postoperative GKRS for the treatment of benign meningiomas, we performed a matched cohort retrospective study.

## Methods

### Patient selection

**Fig. 1:**
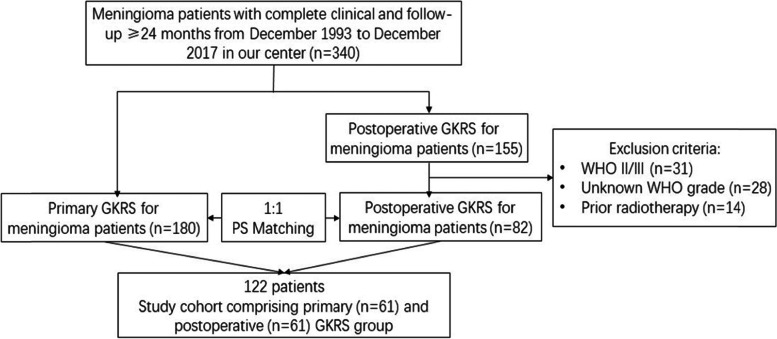
Flowchart showing the patient selection process

The medical records of meningioma patients who underwent GKRS in our center between December 1993 and December 2017 were retrospectively reviewed. Three hundred forty patients had complete clinical data and sufficient follow-up (≥ 24 months) in our center, including primary (*n* = 185) and postoperative GKRS (*n* = 155), respectively. Patients in the postoperative GKRS group were matched to those in the primary GKRS group. The patient selection process was shown in Fig. 1. The indications of primary GKRS for meningiomas in this study included: 1) asymptomatic or mildly symptomatic tumors; 2) unfavorable locations for surgical resection (such as skull base tumors and those close to vascular or neural structures); 3) because of comorbidities or an advanced age; 4) patients’ preference. The indication of postoperative GKRS for meningiomas in this study was the residual or recurrent meningiomas after surgery.

### Radiological and clinical evaluations

All patients were routinely followed up with radiological and clinical evaluation every 6 months for the first 3 years and thereafter yearly. The clinical PFS was defined as the time interval between GKRS and the time of developing new or worsened neurological symptoms or signs in this study. Tumor shrinkage was defined as at least 10% shrinkage in at least one of the meningioma diameters. Tumor progression was defined as tumor enlargement at least 10% in at least one of the meningioma diameters [12]. Distant failure was defined as a new meningioma outside the prior irradiated area appearing during follow-up MRI [12]. Tumor volume was calculated using the following formula: V = anteroposterior diameter × horizontal diameter × vertical diameter × π/6 [13].

### Radiosurgical techniques

The GKRS treatment was performed using Leksell Gamma Knife (Elekta Instruments, Inc, Stockholm, Sweden). Before April 2014, all of the patients were treated with Gamma Knife Unit B. Perfexion Unit was used from April 2014 to the present. After local anesthesia, Leksell stereotactic frame G was placed, then stereotactic MRI with contrast was performed to obtain precise imaging data of tumors for target delineation. GKRS treatment plan was designed by experienced neurosurgeons, medical physicists and radiation oncologists. All of patients in this study underwent single session of GKRS.

### Statistical analysis

The normality of distribution of continuous variables was assessed with Kolmogorov–Smirnov test. Continuous variables with normal distribution were reported as mean (± SEM). Variables not normally distributed were analyzed using median and interquartile ranges (IQR). homogeneity of variance in continuous variables was tested by F test. Categorical variables were presented as frequency and percentage. An independent-sample t-test was used to compare means of continuous variables with normal distribution. The Wilcoxon rank-sum test was used when continuous variables were not normally distributed. Chi-square tests were used for the statistical analysis of categorical variables. Patients in the postoperative GKRS group were matched to those in the primary GKRS group, using propensity scores, in a 1:1 ratio based on sex, age, max tumor diameter, preexisting peritumoral edema (PTE), GKRS margin dose and tumor location (divided as skull base tumors and non-skull base tumors). The “nearest neighbor” method was used for propensity matching with a caliper of 0.10. Log-rank test statistics and a step forward likelihood ratio method of Cox proportional hazard models were used to perform univariate analysis and multivariate analysis respectively. Kaplan–Meier curves were plotted for progression-free survival (PFS), clinical PFS and proportion with radiation-induced edema. Probability values < 0.05 were considered statistically significant. IBM’s SPSS version 26.0 (IBM) with R essential package (R 3.5.0) was used for statistical analysis.

## Results

### Baseline and treatment characteristics

After matching on propensity scores, the study population consisted of 122 patients, including primary (*n* = 61) and postoperative GKRS groups (*n* = 61) (Fig. 1). Each group consisted of 17 (27.9%) males. The mean age was 46.8 and 47.6 years in the primary and postoperative GKRS group. There were 22 (36.1%) and 21 (34.4%) non-skull base tumors in primary and postoperative GKRS group respectively. In the primary GKRS group, the median baseline max tumor diameter, baseline tumor volume, margin dose, max dose and prescription isodose were 34.8 mm, 13.1 ml, 13.0 Gy, 32.5 Gy and 40%, respectively. In the operative GKRS group, the median baseline max tumor diameter, baseline tumor volume, margin dose, max dose and prescription isodose were 35.3 mm, 12.5 ml, 13.0 Gy, 33.0 Gy and 40%, respectively. More symptomatic tumors were in the postoperative GKRS group (*p* < 0.026). Other baseline and treatment characteristics were similar in the two groups. (Table 1).

**Table 1:** Comparison of the baseline and treatment characteristics between the primary and postoperative GKRS group

Characteristic	Primary GKRS	Postoperative GKRS	*P* Value	Combined group
No. of patients	61	61	NA	122
Male, n (%)	17 (27.9)	17 (27.9)	> 0.999	34 (27.9)
Mean age, years	46.8 ± 1.5	47.6 ± 1.3	0.688	47.2 ± 1.0
Non-skull base tumors, n (%)	22 (36.1)	21 (34.4)	0.850	43 (35.2)
Mean max diameter, mm	34.8 ± 1.5	35.3 ± 1.5	0.819	35.0 ± 1.1
Tumor volume, median, (IQR), ml	13.1 (5.8–18.8)	12.5 (5.7–21.1)	0.860	12.8 (5.7–21.0)
Preexisting PTE, n, (%)	6 (9.8)	5 (8.2)	0.752	11 (9.0)
FU duration, median, (IQR), months	73.8 (42.0–92.5)	68.5 (37.0–112.6)	0.961	72.7 (37.5–103.6)
Symptomatic tumors, n, (%)	31 (50.8)	43 (70.5)	0.026^※^	74 (60.7)
CN dysfunction	24	24	NA	48
I	0	1	NA	1
II	6	16	NA	22
III/IV/VI	5	5	NA	10
V	11	2	NA	13
VII	4	1	NA	5
VIII	6	3	NA	9
IX	1	1	NA	2
X	1	1	NA	2
Headache	6	16	NA	22
Seizures	3	2	NA	5
Vomiting	0	2	NA	2
Extremity numbness	1	3	NA	4
Extremity weakness	2	3	NA	5
Ataxia	2	3	NA	5
Tumor location	NA	NA	NA	NA
Foramen magnum	0	1	NA	1
Frontobasal	2	3	NA	5
Tentorium	2	4	NA	6
Convexity	2	5	NA	7
CPA	9	11	NA	20
Falx/parasagittal	19	15	NA	34
Intraventricular	1	1	NA	2
Orbital	1	3	NA	4
Parasellar/cavernous sinus	12	4	NA	16
Petroclival	5	1	NA	6
Sphenoidal	7	8	NA	15
Suprasellar	1	5	NA	6
Margin dose, median, (IQR), Gy	13.0 (12.0–13.0)	13.0 (12.0–14.0)	0.522	13.0 (12.0–13.5)
Maximum dose, median, (IQR), Gy	32.5 (30.0–34.2)	33.0 (30.0–35.0)	0.369	32.5 (30.0–35.0)
Prescription isodose, median, (IQR), %	40 (38–40)	40 (35–43)	0.576	40.0 (36.3–40.8)

Data are expressed as number, mean ± SEM, median and IQR, or percentage

*Abbreviations*: *FU* follow up, *GKRS* gamma knife radiosurgery, *PTE* peritumoral edema, *IQR* interquartile range, *CPA* cerebellopontine angle

^※^Statistically significant (*P* < 0.05)

### Surgical complications in the postoperative GKRS group

In the postoperative GKRS group, these patients underwent surgical resection in different hospitals. Among them, 43 patients underwent surgical resection in our hospital. There were 40 (65.6%) and 21 (34.4%) patients with residual and recurrence tumors after surgery. Fifteen (24.6%) patients presented with new or worsened neurological symptoms or signs, including cranial nerve (CN) disfunction (*n* = 8), hemiparesis (*n* = 1), extremity weakness (*n* = 2), lower extremity numbness (*n* = 1), ataxia (*n* = 2), seizures (*n* = 3) and memory decline (*n* = 1) (Table 2).

**Table 2:** Clinical neurological symptoms or signs after surgical resection in postoperative GKRS group

Outcomes of neurological symptoms or signs	Postoperative GKRS, *n* = 61
Improvement	23
Stable	23
New or worsened, n (%)	15 (24.6)
CN dysfunction	8
I	1
II	2
III/IV/VI	5
V	1
VII	2
VIII	1
IX	1
Hemiparesis	1
Extremity weakness	2
Lower extremity numbness	1
Ataxia	2
Seizures	3
Memory decline	1

*Abbreviations*: *GKRS* gamma knife radiosurgery, *CN* cranial nerve

### Radiological outcomes after GKRS

**Fig. 2:**
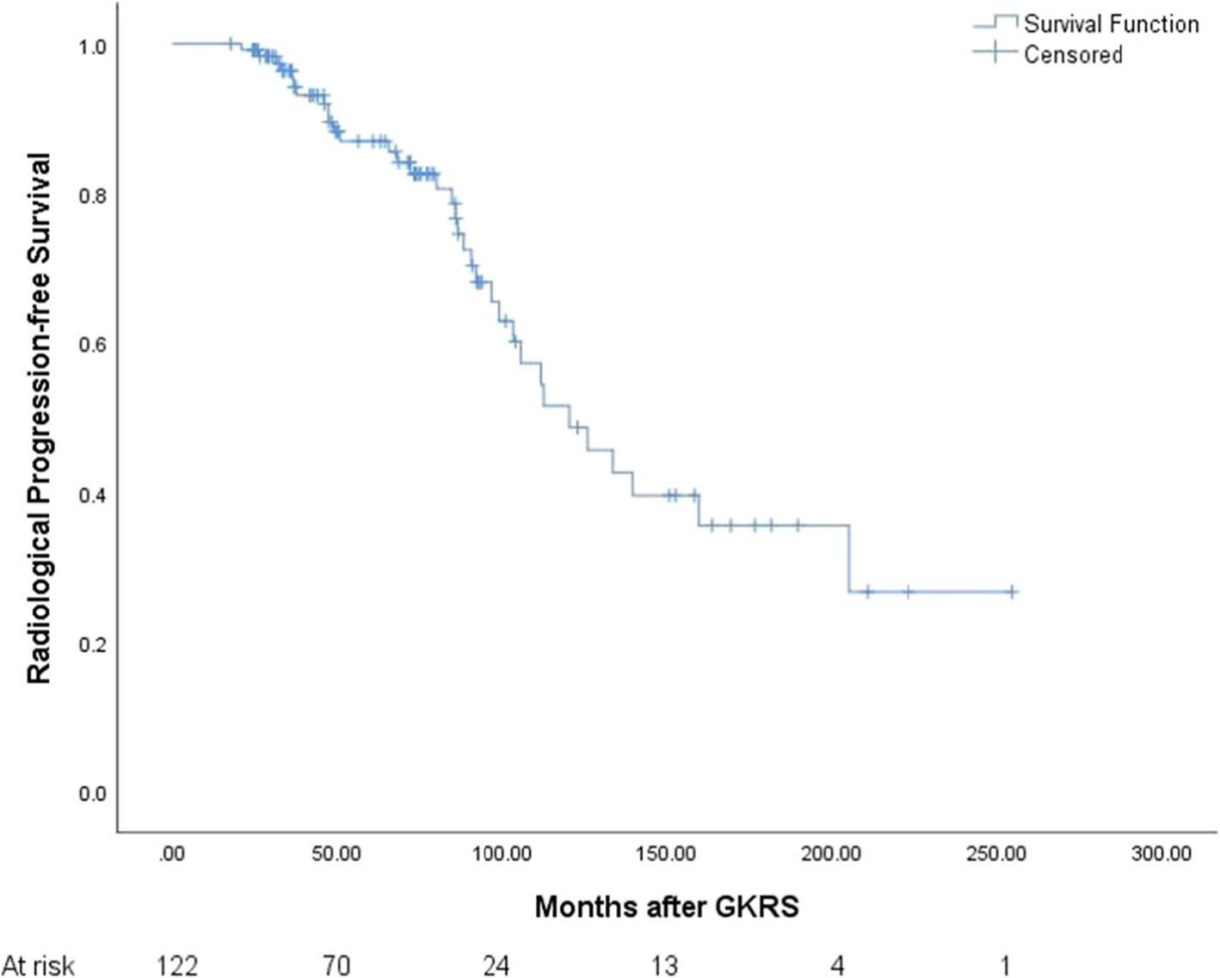
Kaplan–Meier curve of radiological PFS in combined group. The PFS was 100%, 93%, 87%, and 49%, at 1, 3, 5, and 10 years

**Fig. 3:**
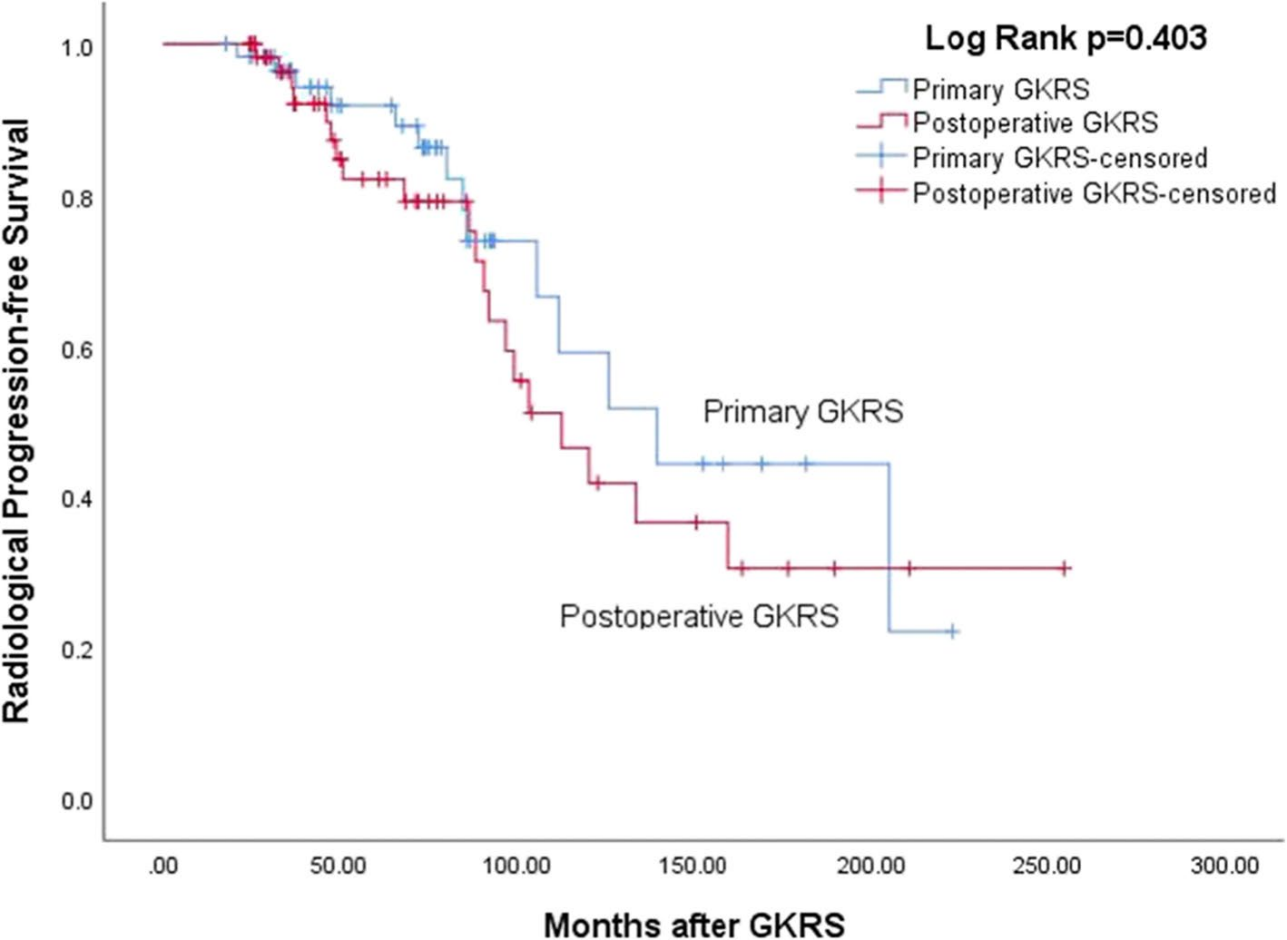
Kaplan–Meier curve comparing radiological PFS between primary versus postoperative GKRS group. The radiological PFS rates were similar between the 2 matched group (*p* = 0.403)

In the combined group, 34 patients (27.9%) occurred radiological progression after a median follow-up of 72.7 (range, 24.2–254.5) months. The median time to radiological progression was 85.1 (range, 20.7–205.1) months. The radiological PFS was 100%, 93%, 87%, and 49%, at 1, 3, 5, and 10 years respectively (Fig. 2). Follow-up MRI confirmed radiological progression in 14 (23.0%) and 20 (32.8%), distant failure in 5 (8.2%) and 8 (13.1%), tumor control in 47 (77.0%) and 41 (67.2%) patients in primary and postoperative GKRS group respectively (Table 3). There was no significant difference in radiological progression (*p* = 0.403) (Fig. 3) and distant failure (*p* = 0.480) between primary and postoperative GKRS groups by log-rank test. For further treatment, in the primary GKRS group, 12 patients underwent repeat GKRS for tumor radiological progression, 1 patient with tumor located at convexity underwent surgical resection, another patient located at CPA underwent surgical resection and repeated GKRS for residual tumor. In the postoperative GKRS group, 15 patients underwent repeat GKRS for tumor radiological progression, 2 patients located at falx/parasagittal underwent surgical resection, 1 patient located at petroclival underwent surgical resection and repeated GKRS for residual tumor, another 2 patients were lost to follow-up.

**Table 3:** GKRS treatment outcomes in the primary GKRS, postoperative GKRS and combined group

Outcomes	Primary GKRS, *n* = 61, (%)	Postoperative GKRS, *n* = 61, (%)	*P* value	Combined group, *n* = 122, (%)
Radiological outcomes	NA	NA	NA	NA
Tumor control	47 (77.0)	41 (67.2)	NA	88 (72.1)
Tumor shrinkage	32	31	NA	63 (51.6)
Stable tumor	15	10	NA	25 (20.5)
Progression	14 (23.0)	20 (32.8)	0.403	34 (27.9)
Distant failure	5 (8.2)	8 (13.1)	0.480	13 (10.7)
GKRS related adverse effects	16 (26.2)	9 (14.8)	0.138	25 (20.5)
Radiation-induced edema	14 (23.0)	7 (11.5)	0.102	21 (17.2)
Symptomatic	5	2	NA	7
Asymptomatic	9	5	NA	14
Clinical outcomes	NA	NA	NA	NA
Improvement	11	7	NA	18
Stable	33	40	NA	73
Clinical progression	17 (27.9)	14 (23.0)	0.336	31 (25.4)
CN dysfunction	10	6	NA	16
I	1	0	NA	1
II	4	3	NA	7
III/IV/VI	6	1	NA	7
V	4	2	NA	6
VII	1	1	NA	2
VIII	0	1	NA	1
Headache	5	4	NA	9
Seizures	4	2	NA	6
Extremity numbness	2	1	NA	3
Extremity weakness	1	2	NA	3
Memory decline	1	1	NA	2

**Fig. 4:**
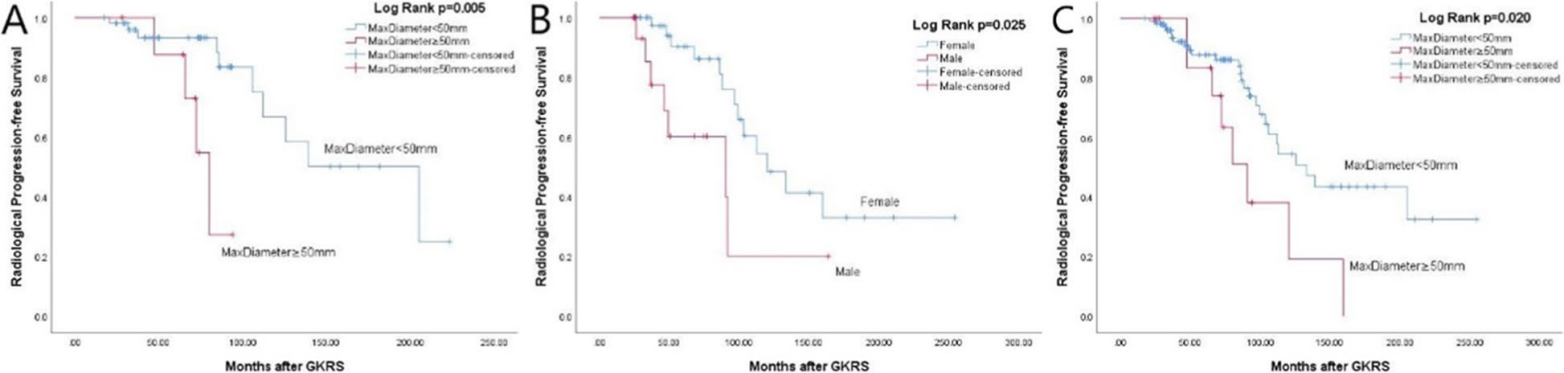
**A**, Kaplan–Meier curve of radiological PFS of max tumor diameter ≥ 50 mm VS < 50 mm in primary GKRS group (*p* = 0.005). **B**, Kaplan–Meier curve of radiological PFS of gender in postoperative GKRS group (*p* = 0.025). **C**, Kaplan–Meier curve of radiological PFS of max tumor diameter ≥ 50 mm VS < 50 mm in combined group (*p* = 0.020)

In univariate analysis, max tumor diameter (≥ 50 mm) (*p* = 0.005) (Fig. 4A) and tumor margin dose (*p* = 0.021) were significantly associated with tumor radiological progression in the primary GKRS group; male (*p* = 0.025) (Fig. 4B) and max tumor diameter (≥ 50 mm) (*p* = 0.020) (Fig. 4C) were significantly associated with tumor radiological progression in postoperative GKRS and combined group respectively. In multivariate analysis, only max tumor diameter (≥ 50 mm) (hazard ratio (HR) = 5.650, 95% confidence interval (CI) = 1.450–22.017, *p* = 0.013) and male (HR = 2.824, 95% CI = 1.099–7.252, *p* = 0.031) were significantly related to tumor radiological progression in primary and postoperative GKRS group respectively (Table 4).

**Table 4:** Univariate and multivariate Cox proportional hazards regression analyses for radiological progression radiation-induced edema and clinical progression in the primary GKRS, postoperative GKRS and combined group

Characteristic	Radiological progression				Radiation-induced edema				Clinical progression			
	Univariate	Multivariate			Univariate	Multivariate			Univariate	Multivariate		
	*p*	HR	CI	*P*	*p*	HR	CI	*P*	*p*	HR	CI	*p*
Primary GKRS												
Age ≥ 55 years	0.602	NA	NA	NA	0.393	NA	NA	NA	0.955	NA	NA	NA
Male	0.462	NA	NA	NA	0.382	NA	NA	NA	0.748	NA	NA	NA
Max diameter ≥ 50 mm	**0.005**^**※**^	**5.650**	**1.450–22.017**	**0.013**^**※**^	0.982	NA	NA	NA	0.152	NA	NA	NA
Tumor volume ≥ 10 ml	0.081	NA	NA	0.198	0.652	NA	NA	NA	0.137	NA	NA	NA
Non-skull base tumors	0.180	NA	NA	NA	** < 0.001**^**※**^	**7.935**	**2.118–29.727**	**0.002**^**※**^	**0.039**^**※**^	NA	NA	0.146
Preexisting PTE	0.417	NA	NA	NA	** < 0.001**^**※**^	**3.572**	**1.012–12.611**	**0.048**^**※**^	**0.003**^**※**^	**6.597**	**1.598–27.224**	**0.009※**
Margin dose ≥ 13 Gy	**0.021**^**※**^	NA	NA	0.075	0.353	NA	NA	NA	0.187	NA	NA	NA
Maximum dose < 30 Gy	0.803	NA	NA	NA	0.565	NA	NA	NA	0.882	NA	NA	NA
Postoperative GKRS												
Age ≥ 55 years	0.161	NA	NA	NA	0.432	NA	NA	NA	0.468	NA	NA	NA
Male	**0.025**^**※**^	**2.824**	**1.099–7.252**	**0.031**^**※**^	0.449	NA	NA	NA	0.939	NA	NA	NA
Max diameter ≥ 50 mm	0.310	NA	NA	NA	0.080	NA	NA	NA	**0.027**^**※**^	NA	NA	NA
Tumor volume ≥ 10 ml	0.533	NA	NA	NA	0.840	NA	NA	NA	0.440	NA	NA	NA
Non-skull base tumors	0.103	NA	NA	NA	0.667	NA	NA	NA	0.870	NA	NA	NA
Preexisting PTE	0.303	NA	NA	NA	0.458	NA	NA	NA	0.372	NA	NA	NA
Margin dose ≥ 13 Gy	0.468	NA	NA	NA	0.326	NA	NA	NA	0.770	NA	NA	NA
Maximum dose < 30 Gy	0.064	NA	NA	0.300	0.239	NA	NA	NA	0.308	NA	NA	NA
Postoperative recurrence	0.359	NA	NA	NA	0.667	NA	NA	NA	0.675	NA	NA	NA
Combined group												
Age ≥ 55 years	0.135	NA	NA	NA	0.267	NA	NA	NA	0.736	NA	NA	NA
Male	0.327	NA	NA	NA	0.853	NA	NA	NA	0.717	NA	NA	NA
Max diameter ≥ 50 mm	**0.020**^**※**^	NA	NA	NA	0.274	NA	NA	NA	**0.007**^**※**^	**2.896**	**1.280–6.553**	**0.011※**
Tumor volume ≥ 10 ml	0.125	NA	NA	NA	0.838	NA	NA	NA	0.089	NA	NA	0.277
Non-skull base tumors	0.618	NA	NA	NA	**0.003**^**※**^	**3.611**	**1.489–8.760**	**0.005**^**※**^	0.137	NA	NA	NA
Preexisting PTE	0.746	NA	NA	NA	**0.026**^**※**^	**3.571**	**1.167–10.929**	**0.026**^**※**^	0.163	NA	NA	NA
Margin dose ≥ 13 Gy	0.351	NA	NA	NA	0.198	NA	NA	NA	0.402	NA	NA	NA
Maximum dose < 30 Gy	0.262	NA	NA	NA	0.453	NA	NA	NA	0.664	NA	NA	NA
Primary GKRS	0.403	NA	NA	NA	0.102	NA	NA	NA	0.336	NA	NA	NA

*Abbreviations*: *GKRS* gamma knife radiosurgery, *PTE* peritumoral tumor edema, *NA* not available, *CI* confidential interval, *HR* hazards ratio

^※^Statistically significant (*P* < 0.05)

Boldface type indicates statistical significance

### Clinical outcomes after GKRS

**Fig. 5:**
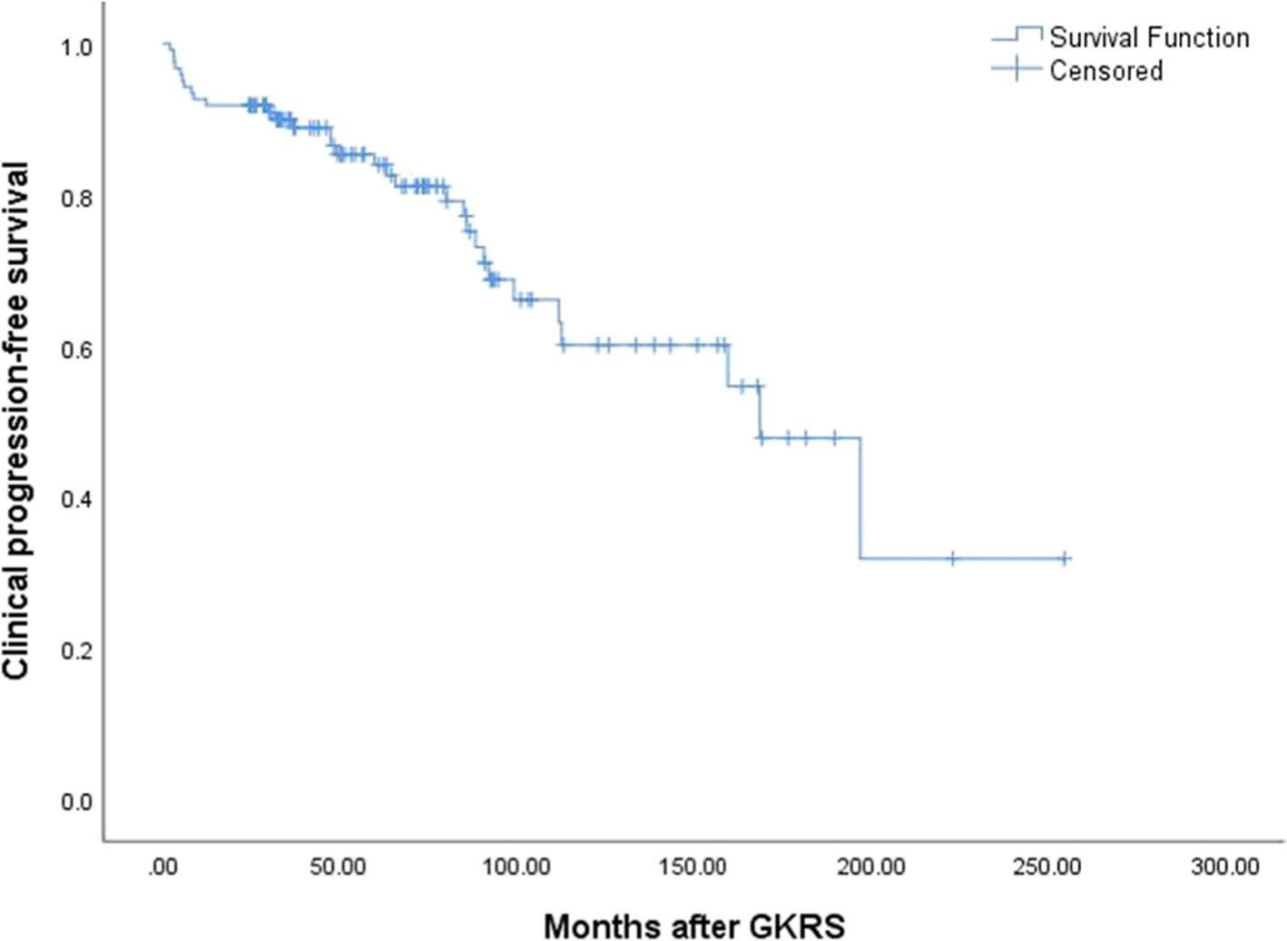
Kaplan–Meier curve of clinical PFS in combined group. The clinical PFS was 92%, 89%, 84%, and 60%, at 1, 3, 5, and 10 years

After GKRS, neurological symptoms or signs improved in 18 patients, and remained stable in 73 patients. Thirty-one patients (25.4%) occurred clinical progression (Table 3). The median time to clinical progression was 48.9 (range, 2.0–196.9) months. Of the 31 patients, 19 patients (61.3%) with neurological symptoms or signs deterioration were due to tumor radiological progression, 7 patients (22.6%) were due to symptomatic radiation-induced edema, 1 patient was due to distant failure, another 4 patients developed CN dysfunction (*n* = 2), memory decline (*n* = 1) or seizures (*n* = 1) without tumor progression and PTE might be caused by GKRS. The clinical PFS was 92%, 89%, 84%, and 60%, at 1, 3, 5, and 10 years (Fig. 5). The log-rank test showed no significant difference in clinical PFS (*p* = 0.336) between primary and postoperative GKRS groups.

**Fig. 6:**
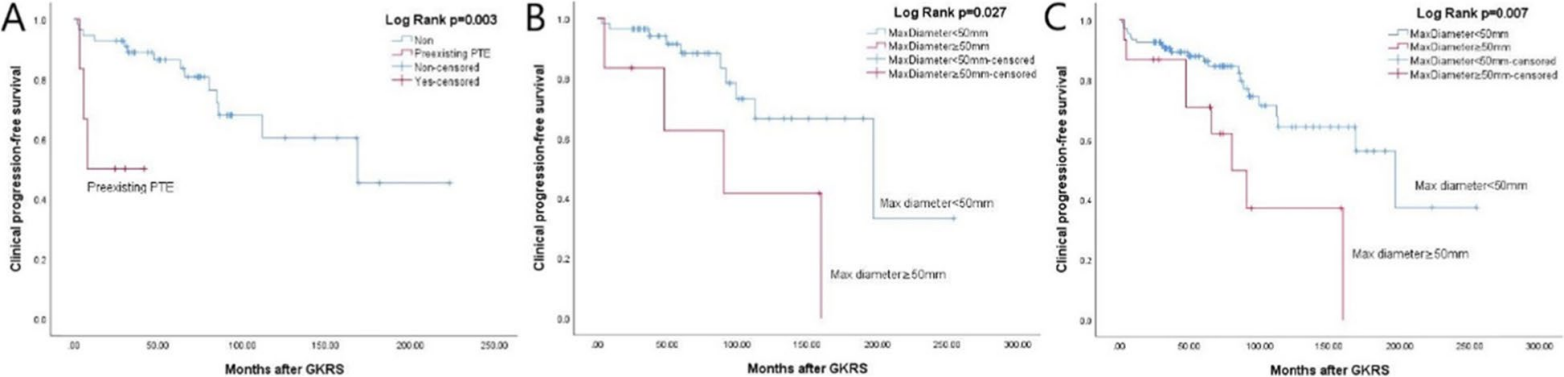
**A**, Kaplan–Meier curve of clinical PFS of preexisting PTE in primary GKRS group (*p* = 0.003). **B**, Kaplan–Meier curve of clinical PFS of max tumor diameter ≥ 50 mm VS < 50 mm in postoperative GKRS group (*p* = 0.027). **C**, Kaplan–Meier curve of clinical PFS of max tumor diameter ≥ 50 mm VS < 50 mm in combined group (*p* = 0.007)

In univariate analysis, non-skull base tumors (*p* = 0.039) and preexisting PTE (*p* = 0.003) (Fig. 6A) were significantly associated with clinical PFS in the primary GKRS group; max tumor diameter (≥ 50 mm) was significantly associated with clinical PFS in postoperative GKRS (*p* = 0.027) (Fig. 6B) and combined groups (*p* = 0.007) (Fig. 6C). In multivariate analysis, only preexisting PTE (HR = 6.597, 95% CI = 1.598–27.224,* p* = 0.009) and max tumor diameter (≥ 50 mm) (HR = 2.896, 95% CI = 1.280–6.553, *p* = 0.011) were significantly related to clinical PFS in primary GKRS and combined groups respectively (Table 4).

### GKRS related adverse effects

**Fig. 7:**
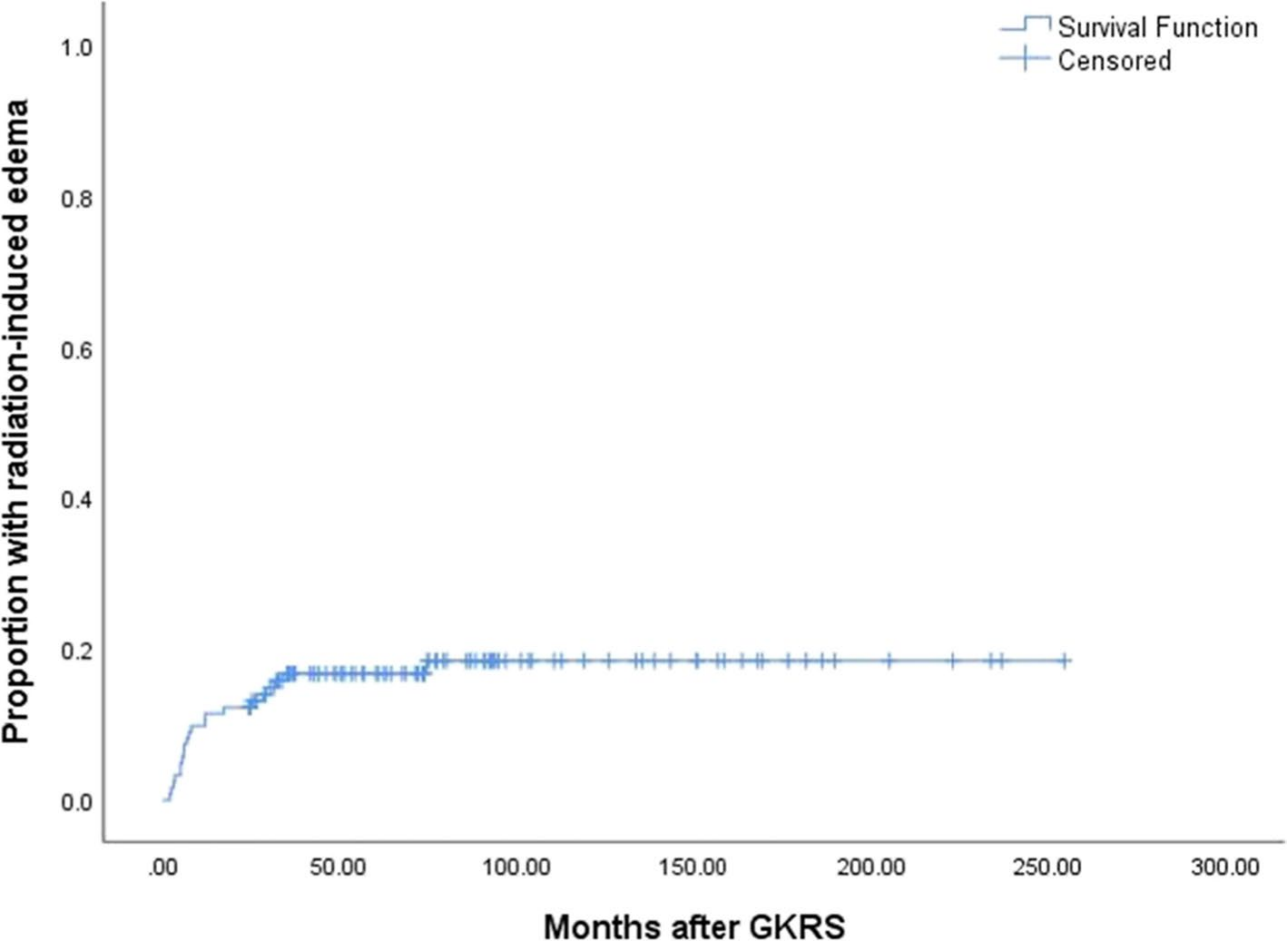
Kaplan–Meier curve of proportion with radiation-induced edema in combined group

Twenty-five patients (20.5%) developed GKRS related adverse effects, including CN dysfunction (*n* = 2), memory decline (*n* = 1) or seizures (*n* = 1), and radiation-induced edema (*n* = 21) (Fig. 7). The median time to GKRS related adverse effects was 8.0 (2.0–74.5) months. There was no significant difference in GKRS related adverse effects (*p* = 0.138) between primary and postoperative GKRS groups by log-rank test. The most common GKRS related adverse effect was radiation-induced edema. The median time to radiation-induced edema was 7.3 (range, 2.0–74.5) months. Seven patients were symptomatic edema, including 5 and 2 patients in the primary and postoperative GKRS group. Among of them, 1 patient presented with severe radiation-induced edema and necrosis underwent surgical resection in the postoperative GKRS group. In the primary GKRS group, 1 patient underwent surgical resection for severe radiation-induced edema and necrosis, 1 patient underwent surgery for seizure due to severe radiation-induced edema. Another 4 patients with symptomatic edema were under control by oral corticosteroids. Fourteen patients with asymptomatic edema were under observation. (Table 3).

**Fig. 8:**
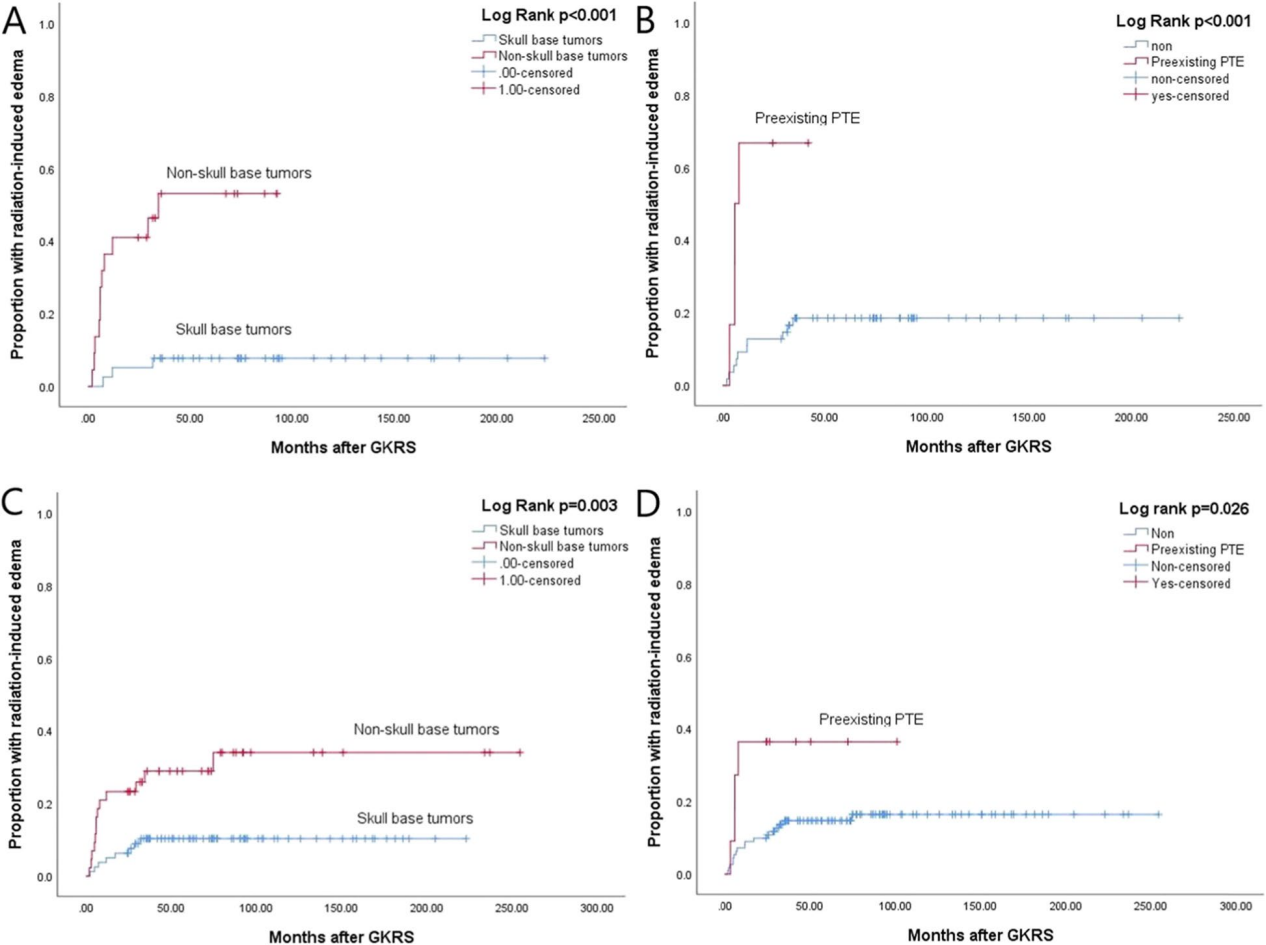
**A**, Kaplan–Meier curve of proportion with radiation-induced edema of non-skull base tumors VS skull base tumors in primary GKRS group (*p* < 0.001). **B**, Kaplan–Meier curve of proportion with radiation-induced edema of preexisting PTE in primary GKRS group (*p* < 0.001). **C**, Kaplan–Meier curve of proportion with radiation-induced edema of non-skull base tumors VS skull base tumors in combined group (*p* = 0.003). **D**, Kaplan–Meier curve of proportion with radiation-induced edema of preexisting PTE in combined group (*p* = 0.026)

In univariate analysis, non-skull base tumors (*p* < 0.001) (Fig. 8A) and preexisting PTE (*p* < 0.001) (Fig. 8B) were significantly associated with radiation-induced edema in the primary GKRS group; non-skull base tumors (*p* = 0.003) (Fig. 8C) and preexisting PTE (*p* = 0.026) (Fig. 8D) were significantly associated with radiation-induced edema in combined group. In multivariate analysis, non-skull base tumors (HR = 7.935, 95% CI = 2.118–29.727, *p* = 0.002) and preexisting PTE (HR = 3.572, 95% CI = 1.012–12.611, *p* = 0.048) were significantly related to radiation-induced edema in the primary GKRS group. Non-skull base tumors (HR = 3.611, 95% CI = 1.489–8.760, *p* = 0.005) and preexisting PTE (HR = 3.571, 95% CI = 1.167–10.929, *p* = 0.026) were significantly related to radiation-induced edema in combined group. (Table 4).

## Discussion

Although surgical resection is the mainstay in the management of meningiomas, it may not be preferred or advisable for all patients due to unfavorable location, comorbidities or advanced age. However, GKRS can provide an alternative treatment for primary and postoperative residual or recurrent meningiomas.

### Advantages and limitations of surgical resection

Surgical resection has the advantages of rapid tumor removal, relief of symptoms, and accurate pathological diagnosis. However, surgical resection is an invasive method, highly depends on tumor location, and can bring about significant morbidity and mortality. Naslund et al. [14] reported that asymptomatic meningioma patients had a significantly higher rate of thirty-day complication and were less likely to work full time than preoperative status after surgical resection [15]. In a study of 34 patients with skull base meningiomas, surgical morbidities occurred in 10 patients (29.4%), with 1 case of mortality [16]. In the postoperative GKRS group of the current study, 15 patients (24.6%) developed new or worsened neurological symptoms or signs after surgical resection. However, recognizing the important of providing a better quality of life and advanced in neurosurgical care, maximum safe resection with low morbidity and preserving neurological function are current aims of neurosurgeons [17, 18].

### Tumor control and related factors

Recent studies have reported PFS of WHO grade I meningiomas treated with SRS ranged from 85 to 100% (median, 89%), and from 53 to 100% (median 85%) at 5 and 10 years respectively [5]. Hasegawa et al. [7] reported SRS treatment outcomes of 67 benign meningioma patients aged ≥ 65 years, actuarial local tumor rates at 5 and 10 years were 86% and 72%, respectively. Previous surgery was significantly associated with failed local tumor control. In a study by Starke et al. [19], 75 large skull base meningioma (> 8 cm^3^) patients underwent SRS, PFS at 5, and 10 years were 88.6%, and 77.2%, respectively. Presentation with any CN deficit, history of radiotherapy and TV > 14 cm^3^ were covariates associated with tumor progression. In a study by Azar et al. [20], 122 sphenopetroclival meningiomas were treated with GKRS. PFS was 56.6% at 5 years. Younger age and lower tumor volume were the main prognostic factors for PFS. Other risk factors, such as male, tumor margin dose, pre-GKRS Karnofsky Performance Scale score, tumors located in the parasagittal/falx/convexity regions had been reported to be associated with tumor progression in some studies [9, 10, 13]. However, Kim et al. [11] and Ge et al. [13] failed to find any relationship between prior surgery and tumor control after SRS. Therefore, whether primary SRS is preferable to postoperative SRS still remains controversial.

In current study, the median tumor max diameter and tumor volume were 35 mm and 12.8 ml. The PFS was 87%, and 49%, at 5, and 10 years respectively, which was a little lower than studies of Hasegawa et al. [7] and Starke et al. [19], but higher than the study of Azar et al. [20]. Perhaps, the tumor control rate decreased due to the large tumor size in our study. Max diameter ≥ 50 mm was significantly related to tumor radiological progression in thr combined group. The tumor radiological progression rate in the postoperative GKRS group was a little higher than that in the primary GKRS group (32.8% VS 23.0%). However, it did not reach a significant difference by log-rank test (*p* = 0.403). Previous surgery did not adversely affect the tumor control rate in our study. The result was similar to the studies of Kim et al. [11] and Ge et al. [13].

Some studies had investigated staged or multisession SRS for large meningiomas. Marchetti et al. [21] reported 143 patients who underwent multisession SRS. The median prescription dose was 25 Gy delivered in 3 to 5 fractions. The PFS at 5, and 8 years was 93%, and 90%, respectively, higher than our study. However, the short-term median follow-up of 44 months might not be sufficient for meningiomas. Iwai et al. [22] reported outcomes of staged GKRS for 27 patients with large skull base meningiomas. The median tumor diameters and volume were 39.4 mm and 27.5 cm^3^ respectively. PFS was 78%, 70% and 70% at 5, 10 and 15 years, respectively. Several studies reported Hypofractionated stereotactic radiotherapy (HFRT) for large meningiomas [23–26]. Although the number of patients was limited, 2 studies suggested a potential better tumor control rate in patients treated with HSRT. Han et al. [26] reported 70 large meningioma patients (> 10 cm^3^) who underwent GKRS. The HFRT group provided higher PFS rate at 5 years than the single-session GKRS group (92.9% vs. 88.1%), but no difference (*P* = 0.389). The HFRT group experienced a lower complication rate than single-session GKRS group (*P* = 0.017). Another study by Manabe et al. [24] did not find any difference in PFS between 5-fraction HFRT and single-session SRS. Therefore, up to now, the safety and efficacy of HFRT remain uncertain. Higher-level evidence is needed.

### Clinical outcomes and related factors

Previous studies reported the median neurological deterioration rate was 7.4% (range, 0%-13.3%) [5]. In the study of Gupta et al. [27], the actuarial symptom control of GKRS for 117 asymptomatic meningioma patients at 5 and 10 years was 86% and 70%, respectively. In the study of Ge et al. [13], neurological symptoms or signs deteriorated in 7 (5.4%) after GKRS. Tumor volume ≥ 10 cm^3^ and pre-GKRS CN deficit were risk factors associated with neurological symptoms or signs of deterioration. In the current study, 31 patients (25.4%) occurred clinical progression. The reasons of clinical progression consisted of tumor radiological progression, symptomatic radiation-induced edema, distant failure and GKRS. The clinical PFS was 84%, and 60%, at 5, and 10 years. In the combined group, max tumor diameter (≥ 50 mm) was significantly related to clinical PFS in multivariate analysis (*p* = 0.011). There was no significant difference in clinical PFS between primary and postoperative GKRS groups by log-rank test. The neurological symptoms or signs rate in our study was higher than the study of Ge et al. (25.4% VS 5.4%). This was because of larger tumors and long-term follow-up in our study.

### Radiation related adverse effects

Previous studies reported the median SRS related toxicity rate was 8.0% (range, 2.5%-34.6%) [5]. Factors reported to be associated with SRS related toxicity included tumor location in the anterior fossa, nonbasal location, no prior surgery, present pretreatment PTE, higher margin dose, tumor volume > 10 ml, and age > 60 years [7, 28–35]. In the study of Hasegawa et al. [7], the median tumor margin dose and tumor volume were 16 Gy and 4.9 (range, 0.7–22.9) cm^3^ respectively. Mild or moderate adverse events were noted in 9 patients (13.4%). A higher margin dose was significantly related to adverse effects in univariate analysis. In a study of Pollock et al. [9], the median margin dose and tumor volume were 16 Gy and 7.3 cm^3^ respectively. Forty-five patients (11%) developed permanent radiation-related complications. Increasing tumor volume and patients with tumor of the parasagittal/flax/convexity were risk factors associated with radiation-related complications. In the study of Seo et al. [35], the median tumor volume and marginal dose were 4.35 cm^3^ and 14 Gy, respectively. Symptomatic PTE was identified in 36 (8.5%) patients, and the risk factor related to poor PTE was the presence of PTE before GKRS (*P* < 0.001). Permanent complication rate was 4%.

In the current study, 25 patients (20.5%) developed GKRS related adverse effects, including CN dysfunction (*n* = 2), memory decline (*n* = 1) or seizures (*n* = 1), and radiation-induced edema (*n* = 21). The incidence of GKRS related adverse effects in our study was a little higher than other studies. This may be due to many large meningiomas in this study. Radiation-induced edema was the most common radiation-related adverse effect, accounting for 15% to 28% [28, 29, 31, 32, 36–39]. Several risk factors, including parasagittal location, sagittal sinus occlusion, preexisting PTE, tumor volume, radiation dose and hemispheric tumor location had been reported to be associated with PTE [28, 29, 31, 32, 37–39]. In a study of Hasegawa et al. [6], the incidence of symptomatic radiation-induced edema was significantly higher in patients who underwent GKRS as the initial treatment. Fewer prior treatments and low margin dose were significantly associated with radiation-induced edema. In the study of Cai et al. [28], of 182 meningiomas treated with SRS, 45 (24.7%) developed post-SRS PTE. Preexisting PTE and tumor-brain contact interface area were the most significant risk factors for post-SRS PTE. In the current study, non-skull base tumors and preexisting PTE were significantly related to radiation-induced edema in primary GKRS and combined groups in multivariate analysis. No risk factor was found related to radiation-induced edema in postoperative GKRS group.

### Study limitations

This is a retrospective cohort study of primary versus postoperative GKRS for intracranial benign meningiomas. Several limitations should be noticed. First, propensity score matching was used to reduce baseline differences between primary GKRS and postoperative GKRS groups, but it can never adjust for unmeasured confounder factors. Therefore, propensity score matching can never result in definitive conclusions about cause-effect relationships. Second, treatment and selection biases cannot be ignored in a retrospective study. Third, in the primary GKRS group, these patients did not undergo surgical resection before GKRS and were lack of pathological information, which probably consisted of some WHO II/III grade meningiomas, and might underestimate the tumor control rate. Four, the relatively small number of patients in this study might lead to the limited statistical power. Finally, the GKRS instrument was upgraded in 2014, which might influence treatment strategy.

## Conclusions

In this long-term retrospective matched cohort study, we found that primary GKRS could provide similar radiological and clinical outcomes, as well as similar complication rates compared with postoperative GKRS. The tumor control rate was 72.1%. The clinical progression rate was 25.1%. GKRS related adverse effects occurred in 20.5%. Because of many large tumors in this study, the 10-year PFS rate was 49%, which was relatively low. Therefore, further study is needed to improve the tumor control rate for large tumors. In conclusion, for selective benign meningioma patients (asymptomatic or mildly symptomatic tumors; unfavorable locations for surgical resection; comorbidities or advanced age), GKRS could be an alternative primary treatment.

## Data Availability

The datasets generated and/or analysed during the current study are not publicly available due to part of the data in the study related to other studies, but are available from the corresponding author on reasonable request.
